# Circulating plasma factors involved in rejuvenation

**DOI:** 10.18632/aging.103933

**Published:** 2020-11-16

**Authors:** Jae Sook Kang, Yong Ryoul Yang

**Affiliations:** 1 Aging Research Center, Korea Research Institute of Bioscience and Biotechnology, Daejeon, Republic of Korea; 2 Department of Functional Genomics, KRIBB School of Bioscience, Korea University of Science and Technology (UST), Daejeon, Republic of Korea

**Keywords:** aging, plasma proteins, rejuvenation, young blood

## Abstract

Aging is defined as a time-dependent functional decline that occurs in many physiological systems. This decline is the primary risk factor for prominent human pathologies such as cancer, metabolic disorders, cardiovascular disorders, and neurodegenerative diseases. Aging and age-related diseases have multiple causes. Parabiosis experiments, in which the circulatory systems of young and old mice were surgically joined, revealed that young plasma counteracts aging and rejuvenates organs in old mice, suggesting the existence of rejuvenating factors that become less abundant with aging. Diverse approaches have identified a large number of plasma proteins whose levels differ significantly between young and old mice, as well as numerous rejuvenating factors that reverse aged-related impairments in multiple tissues. These observations suggest that increasing the levels of key rejuvenating factors could promote restorative biological processes or inhibit pathological degeneration. Inspired by such findings, several companies have begun selling “young blood transfusions,” and others have tested young plasma as a treatment for Alzheimer’s disease. Here, we summarize the current findings regarding rejuvenating factors.

## INTRODUCTION

The blood is a circulating representation of all body tissues and hence carries a plethora of information about the overall pathophysiology of an individual. Age-related changes in the blood have been investigated by metabolomics or proteomics, with the goal of understanding the mechanisms of aging and age-related pathogenesis, and also to identify candidate diagnostic and prognostic biomarkers for diseases [1–4]. Longitudinal metabolomics data from human plasma have revealed that plasma metabolite levels are influenced by aging, and that numerous metabolites are correlated with age [1, 5]. Using the SomaScan aptamer technology, two studies identified a conserved aging signature in human plasma proteomic data [2, 3]. These observations offer a deeper understanding of the aging process and provide new insight into the molecular mechanisms underlying human health and aging.

Changes in plasma proteins contribute to the aging process, as well as age-related diseases. Parabiosis models, aimed at elucidating the effect of young or old blood on aging, revealed that young blood rejuvenates aged tissues in multiple organs, including brain, heart, pancreas, bone, skeletal muscle [6–10]. These studies strongly support the notion that both pro-aging and rejuvenating factors are present in the circulation, and a wide range of efforts are underway to identify these factors. Initial studies in parabiosis models identified GDF11 as a molecule capable of rejuvenating cerebral, cardiac, and skeletal muscle functions [7, 11, 12]. Similarly, the C-C motif chemokine 11 (CCL11) and β2 microglobulin (B2M) negatively regulate neurogenesis and cognitive function in the hippocampus. In addition, several plasma proteins have been identified as rejuvenating factors that provide beneficial effects in diverse tissues. Based on the identification of these circulating proteins, anti-aging startups have started to develop new therapies targeting age-related diseases [13].

**Figure 1:**
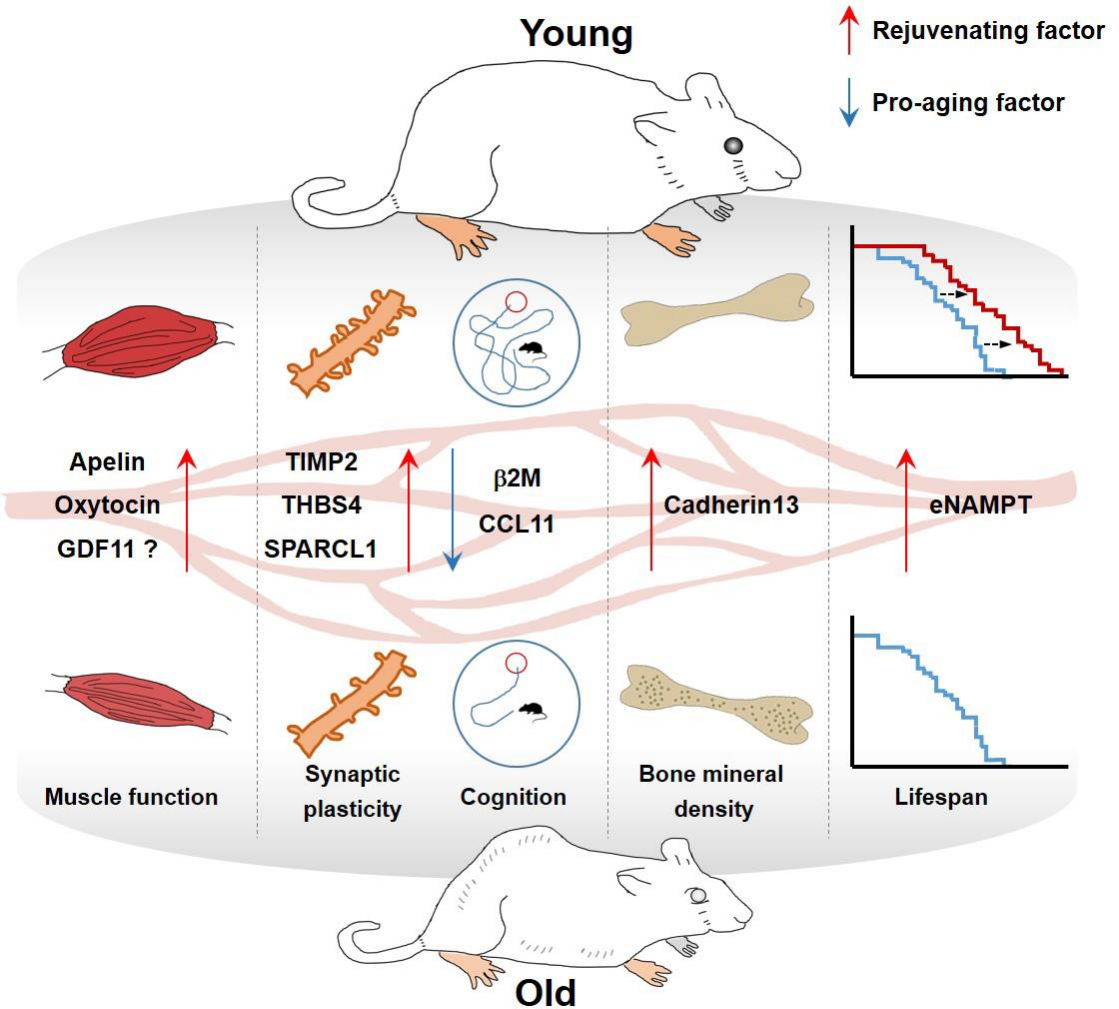
**Effect of blood factors on organs.**

The discovery and validation of rejuvenating factors have been investigated so far with diverse approaches. In this review, we highlight the potential roles of selected plasma proteins that are present at different levels between young and old blood, and describe the results of many fascinating studies reporting pro-aging or rejuvenating factors in the blood (Table 1 and Figure 1).

**Table 1:** Summary of circulating plasma factors involved in rejuvenation.

**Circulating factor**	**Plasma levels in aged mice**	**Class**	**Functions**	**References**
Apelin	Decline	Hormone (Rejuvenating factor)	• Extends the murine healthspan and promotes mitochondriogenesis and protein synthesis	Vinel et al., *Nature Medicine* (2018)
			• Reverses age-associated muscle wasting and delays stress-induced cellular senescence	Rai et al., *Cell Reports* (2017)
β2-microglobulin	Increase	component of MHC class I molecules (Pro-aging factor)	• Impairs hippocampus-dependent cognitive function and neurogenesis	Smith et al., *Nature Medicine* (2016)
Cadherin13	Decline	cell adhesion protein (Rejuvenating factor)	• Inhibits osteoclast differentiation and ameliorates age-associated bone loss	Yang et al. *Aging* (2020)
CCL11	Increase	Cytokine (Pro-aging factor)	• Reduced synaptic plasticity, and impairment of contextual fear conditioning and spatial learning and memory.	Villeda et al., *Nature* (2011)
GDF11	Decline	Cytokine (Rejuvenating factor)	• Reverses age-related cardiac hypertrophy by suppressing phosphorylation Forkhead transcription factor	Loffredo et al., *Cell* (2013)
	Not verified		• Restores the functionality of the vasculature of the neurogenic niches	Katsimpardi et. al., *Science* (2014)
			• Improves muscle physiology and physical function by improving satellite cell competency	Sinha et al., *Science* (2014)
			• Shows calorie restriction–like phenotype	Katsimpardi et al., *Aging Cell* (2020)
			• Increases bone mass by stimulating the BMP signaling pathway, thereby promoting osteogenic differentiation	Suh et al., *PNAS* (2020)
	Increase	(Pro-aging factor)	• Impairs muscle regeneration	Egerman et al. *Cell Metab* (2015)
			• Impairs liver regeneration	Liu et al. Faseb J (2018)
	Not verified		• Induces skeletal muscle atrophy	Hammers et al. *EMBO Mol Med* (2017)
			• Decrease bone mass	Liu et al. Nat Commun (2016)
eNAMPT	Decline	enzyme	• Improves physical activity and extends mouse lifespan by promoting NAD^+^ biosynthesis	Mitsukuni et al. *Cell Metab*. (2019)
Oxytocin	Decline	hormone	• Prevent skeletal muscle aging by promoting proliferation of myogenic progenitor cells	Elabd et al. *Nat. Comm*. (2014)
SPARCL1	Decline	matricellular extracellular matrix protein	• Elevated spontaneous synaptic responses, synapse density, and dendritic branching; enhancement of evoked neurotransmission	Kathlyn et al. *PNAS* (2019)
TIMP2	Decline	Protease inhibitor	• Increases synaptic plasticity and hippocampus-dependent cognition	Castellano et al. *Nature* (2017)
THBS4	Decline	matricellular extracellular matrix protein	• Increased spontaneous synaptic responses, synapse density, and dendritic branching; enhancement of evoked neurotransmission	Kathlyn et al. *PNAS* (2019)

### Apelin

Apelin, a 13-amino acid peptide, was initially identified as an endogenous ligand that regulates gastric acid production through a G protein–coupled receptor, APJ [14, 15]. The human *APLN* gene encodes a proprotein consisting of 77 amino acid residues, which can be cleaved into a 55–amino acid fragment and then into several active fragments. Apelin-13, which is highly bioactive, is responsible for APJ binding and biological activity [16, 17]. Apelin and APJ are widely expressed in the body and play important roles in many organs. In addition, apelin ameliorates the symptoms of many diseases, including, neurological disorders [18], hypertension [19], metabolic disorders [16], gastrointestinal diseases [20], and hepatic diseases [21]. Interestingly, expression of apelin and APJ in tissues decline with age, and stress-induced senescence downregulates endogenous apelin and APJ [22]. Consistent with this, the circulating levels of apelin decrease with age in mice [23, 24]. Although *Apln*^−/−^ and *Aplnr*^−/−^ mice appear healthy, they undergo accelerated multi-organ aging. Genetic or pharmacological inhibition of apelin-mediated signaling leads to cellular senescence, whereas systemic restoration of apelin attenuates age-related pathologies and extends healthspan in mice [22].

Physical exercise ameliorates age-related muscle wasting [25], and restoration of myokines is the main mechanism by which physical activity prevents muscle wasting. Besse-Patin et al. identified apelin as an exercise-regulated myokine in humans [26]. In particular, they found that 8 weeks of endurance exercise training in obese male subjects upregulated *APLN* expression in skeletal muscle. Consistent with this, exercise stimulates secretion of apelin into the bloodstream in both mice and humans. Plasma apelin levels are associated with sarcopenia, defined as a gradual loss of function and muscle with age [23]. Accordingly, chronic apelin administration in old mice increases muscle mass and function by promoting mitochondriogenesis and protein synthesis. In addition, apelin targets muscle stem cells and increases their regenerative capacity. The phenotypes of *Apln*^−/−^ and *Aplnr*^−/−^ mice support the idea that these factors play critical roles muscle physiology during aging. Collectively, these findings suggest that apelin and its receptor are therapeutic targets for the rejuvenation of aged skeletal muscle [23].

### β2-microglobulin

Beta 2–microglobulin (β2M) is a secretory protein that serves as coreceptor for antigen presentation by major histocompatibility complex (MHC) class I to the immune system [27]. β2M is produced by all nucleated cells and is present in the circulation under normal physiological conditions. β2M has no transmembrane region and contains a distinctive molecular structure called a constant-1 Ig superfamily domain. β2M-deficient mice exhibit a wide variety of immunological aberrations, such as antigen-specific IgG production [28], the catabolism of IgG [29], and greater susceptibility to pathogen infections [30–33].

Serum and plasma β2M levels are elevated in many pathological conditions, including renal disease, immunodeficiency, autoimmune diseases, and tumor burden [34–39]. Accelerated aging also affects the concentration of circulating β2M. In the general population of older adults, serum β2M concentration is a predictor of total mortality [40]. Furthermore, among geriatric inpatients, higher levels of β2M are associated with frailty [41]. High levels of plasma β2M are observed in elderly humans and aged mice [42], and circulating β2M levels are elevated in the younger partners in heterochronic parabiosis experiments [8]. Villeda et al. found that β2M acts as pro-aging factor by contributing to age-related decline in adult neurogenesis and impairments in hippocampal-dependent cognitive functions. Systemic administration of β2M or local injection of the protein into the brain impairs hippocampal-dependent neurogenesis and cognitive functions. Aged *B2m^-/-^* mice exhibit superior cognitive function and neurogenesis in comparison aged WT mice [42]. These observations raise the possibility that targeting β2M could attenuate age-related decline in cognition and regenerative functions in old age.

### Cadherin-13

Cadherin-13 (also known as T-cadherin or H-cadherin) is an atypical member of the cadherin superfamily, whose members are membrane proteins that mediate calcium-dependent cellular adhesion. Cadherin-13 lacks transmembrane and cytoplasmic domains, but rather contains a glycosylphosphatidylinositol moiety that anchors it to the plasma membrane [43, 44]. Thus, cadherin-13 acts by interacting with other membrane-bound molecules to regulate cellular functions. It is highly expressed in developing and adult brain [45], and is known as an ADHD-risk gene [46]. Studies have reported that the cadherin-13 protein inhibits neuronal outgrowth, cell migration, and axon guidance [44, 47–49]. Cadherin-13 is also found in the heart, pancreatic β-cells, liver, and skeletal muscle, and studies have shown that it contributes to regulating stress-induced pathological cardiac remodeling and metabolism through binding to adiponectin and insulin granules [50–52]. Although cadherin-13 is attached to the cell membrane, it is detected in the blood. Reduced levels of circulating cadherin-13 have been associated with coronary artery disease [53]. Plasma proteomic profiling revealed that cadherin-13 is expressed at lower levels in old mice compared to young mice [54]. Cadherin-13 inhibits osteoclast differentiation by blocking the activation of RANKL-associated signaling cascades, and cadherin-13 treatment of aged mice was found to delay age-associated bone loss [54]. These results suggest that cadherin-13 is an age-related bone factor that contributes to osteoporosis or osteopenia during aging, and that it could potentially be used as a novel therapeutic molecule for the treatment of bone loss. However, the underlying molecular mechanisms remain to be elucidated.

### CCL11/Eotaxin-1

CCL11, a chemokine that can bind the chemokine receptors CCR2, CCR3, and CCR5 [55, 56], was first identified as a potent eosinophil chemoattractant. Thus, it participates in multiple inflammatory diseases including allergic reactions, allergic reactions, atopic dermatitis, and inflammatory bowel diseases; accordingly, its serum levels are elevated in each of these diseases [57–59]. In addition to its role in the immune response, CCL11 has multiple functions in diverse tissues and organs. Moreover, CCL11 plays roles in diseases, and has been implicated in cancer, atherosclerosis, neurogenesis, and neurodegeneration, and myocardial diseases [8, 60–63]. Notably, in this regard Villeda et al. showed that the plasma levels of CCL11 increase with aging and contribute to impaired neurogenesis and cognitive function in mice; the reduction in neurogenesis could be prevented by CCL11 neutralizing antibodies [8]. Injection of CCL11 into young mice mimicked hippocampal aging, with a reduction in synapses and an increase in microglial activation [64]. Interestingly, plasma levels of CCL11 are negatively associated with memory function in patients with Alzheimer’s dementia and older adults dwelling in rural communities [65, 66]. Furthermore, plasma CCL11 levels are elevated in patients with psychiatric conditions such as schizophrenia, bipolar disorder, dysthymia, and autism spectrum disorder [67–70]. However, the mechanism by which circulating CCL11 affects neuronal dysfunction is currently not well understood. Several studies have suggested that CCL11 may play a neurotoxic role in the pathophysiology of AD and PD [71, 72]. CCL11 is now considered to be a pro-aging factor that promotes aging-related neuronal dysfunction independent of eosinophil recruitment. Inspired by such findings, a startup called Alkahest has initiated a Phase II clinical trial investigating ALK4290, the first non–plasma-derived product targeting CCL11, for treatment of wet age-related macular degeneration (AMD) and Parkinson’s disease.

### eNAMPT

Nicotinamide adenine dinucleotide (NAD^+^) an essential pyridine nucleotide that is present in all living cells. NAD^+^ is an abundant cofactor that participates in multiple aspects of biological processes, including energy metabolism, DNA repair, gene expression, cellular signaling, and stress response [73, 74]. NAD^+^ levels decrease with age, and that elevated intracellular NAD^+^ levels extend lifespan in model organisms [75–81]. Several studies have confirmed that administration of NMN, an intermediate in NAD^+^ biosynthesis, ameliorates age-related dysfunction [76, 81–83]. Nicotinamide phosphoribosyltransferase (NAMPT), a key enzyme in the biosynthesis of nicotinamide adenine dinucleotide (NAD^+^), converts nicotinamide and 5′-phosphoribosyl-pyrophosphate (PRPP) to nicotinamide mononucleotide (NMN) [84]. Stimulation of NAMPT activity has been proposed as a strategy for preventing and treating age-related diseases [85, 86]. In mammals, NAMPT exists in intracellular (iNAMPT) and extracellular (eNAMPT) forms [87]. In addition to its enzymatic function, eNAMPT (also known as pre–B-cell enhancing factor (PBEF)/visfatin) acts as a cytokine in circulation. Remarkably, Yoshida et al. found that circulating eNAMPT is carried in extracellular vesicles and correlates significantly with age in both mice and humans. In mice, increasing eNAMPT levels via adipose-specific overexpression maintains NAD^+^ levels during aging, prevents age-associated physiological decline, and extends lifespan. eNAMPT carried by EVs is internalized into target cells, resulting in upregulation of NMN/NAD^+^ biosynthesis [88]. Based on these findings, NAD^+^ metabolism has emerged as a potential target for treatment of age-related disorders. Indeed, several clinical studies have been planned or are already ongoing in the US and Europe.

### GDF11

Growth differentiation factor 11 (GDF11), a secreted protein, is also known as bone morphogenetic protein 11 (BMP-11). GDF11 is a member of TGF-β/BMP superfamily that mainly activates Smad and non-Smad signaling pathways via binding to Activin receptor I and II, thereby regulating expression of its target genes [89]. GDF11 was initially synthesized as a precursor protein, and then cleaved by pro-protein convertase subtilisin/kexin type 5 (PCSK5), forming a non-covalent latent complex consisting of an N-terminal inhibitory pro-domain and two disulfide-linked carboxyl-terminal active domains [90, 91]. BMP1/Tolloid matrix metalloproteinase further cleaves the latent complex to generate active GDF11 [92].

Over the past few decades, a series of studies revealed that GDF11 participates in embryonic development by regulating anterior/posterior patterning of the axial skeleton, kidney organogenesis, spinal cord neurogenesis, pancreatic beta-cell differentiation, and retinal formation [93–97]. In addition, it plays a critical role in multiple fundamental homeostatic processes including osteogenesis, myogenesis, neurogenesis, and erythropoiesis [7, 12, 98–101]. Therefore, many studies have demonstrated that GDF11-mediated physiological processes have implications in human development and diseases.

Loffredo et al. hypothesized that anti-aging factors might reverse age-related cardiac hypertrophy, and identified GDF11 as anti-aging factor with antihypertrophic properties [11]. Aptamer-based proteomic analysis revealed several circulating proteins present at different levels between young and old mice. Among these candidates, GDF11 level declined with age and administration of GDF11 reversed age-related cardiac hypertrophy in old mice [11]. In addition to cardiac aging, GDF11 reverses skeletal muscle aging: systemic delivery of GDF11 protein restores genomic integrity in aged muscle stem cells and improves muscle physiology and physical functions [12]. GDF11 also exerts positive effects in the aged brain by promoting vascular remodeling and neurogenesis [7]. In contrast to these observations, however, a series of follow-up studies reported that GDF11 negatively affects aged tissues. In particular, GDF11 inhibits myoblast differentiation in mice, thereby impairing muscle regeneration [100]. Moreover, Hinken et al. reported that GDF11 does not affect outgrowth of muscle stem cells [102]; indeed, systemic overexpression of GDF11 induces skeletal and cardiac muscle atrophy in mice [103]. GDF11 inhibits bone formation, and inhibition of GDF11 functions prevents age-related osteoporosis [99]. Furthermore, it remains unclear whether the levels of circulating GDF11 are related to age. Multiple studies using different detection methods have reported conflicting age-related changes in GDF11 levels. The mature active region of GDF11 is highly similar to that of myostatin (GDF8). Consequently, commercially available ELISAs are available using antibodies with cross-reactivity with myostatin. Egerman et al. found that a previously used GDF11 SOMAmer and GDF11 antibodies bind to both GDF11 and myostatin [11, 12, 100], and GDF11-specific immunoassays revealed that serum GDF11 levels are elevated with age in human and rats [100]. Importantly, using a highly specific LC-MS/MS assay, Schafer et al. found that the levels of GDF11 do not decline in men or women throughout the lifespan and are instead positively associated with frailty and other morbidities [104]. The controversy about the relationship between GDF11 and aging may be caused by differences in GDF11 detection methods, the source of recombinant GDF11 protein, experimental design, or other factors. Although the biology of GDF11 remains controversial, the protein has important implications for physiological and pathological processes related to diagnosis and therapy of human diseases.

### Oxytocin

Oxytocin is a hormone produced mainly in the hypothalamus and secreted by the pituitary gland. Oxytocin plays well-known roles in female reproduction, including uterine contraction and milk ejection. The oxytocin receptor, a typical member of the class I G protein–coupled receptor superfamily, is expressed in a variety of tissues, such as the ovary, testis, adrenals, uterus, mammary glands, bone, brain, liver, and adipose [105]. Because the oxytocin receptor is present on diverse cell types, oxytocin has multiple positive physiological and psychological effects [106]. In particular, it influences a wide array of social behaviors through direct projections to other brain regions such as the nucleus accumbens, olfactory bulb, amygdala, and brain stem [107–110]. Although several studies of postmortem neural tissues have investigated whether aging affects the oxytocin system, some controversy persists regarding the number of oxytonergic cells in the brain of elderly subjects [111, 112]. In addition, reduced levels of oxytocin have been detected in postmenopausal women with osteoporosis [113]. Consistent with these observations, ovariectomized mice and rats have significantly lower plasma oxytocin levels than sham-operated mice. Oxytocin enhances osteoblast differentiation, and supplementation of oxytocin reverses bone loss induced by ovariectomy in rodents [114]. Interestingly, circulating oxytocin levels decline with age in rhesus macaques and mice [115, 116]. Elabd et al. [116] suggested that this age-related decline in oxytocin contributes to defects in muscle regeneration. They found that oxytocin rejuvenates muscle stem cells by promoting their proliferation after muscle injury. Furthermore, aged *Oxt^-/-^* mice exhibit premature sarcopenia. Because several lines of evidence have revealed that oxytocin improves social deficits associated with various psychiatric disorders, numerous clinical trials have investigated the effect of this protein on social dysfunction [117]. In addition to improvement of social behavioral dysfunction, oxytocin and the oxytocin-mediated signaling pathway represent new clinical targets for rejuvenation of aged skeletal muscle.

### TIMP2

Tissue inhibitor of metalloproteinases (TIMPs) are a family of secretory proteins consisting of four members: TIMP-1, -2, -3, and -4. TIMPs are endogenous inhibitors of metalloproteinases, including the matrix metalloproteinases (MMPs), a disintegrin and metalloproteinases (ADAMs), and ADAMs with thrombospondin motifs (ADAMTSs) [118]. Generally, TIMPs participate in extracellular matrix (ECM) catabolism, which is essential for many biological processes such as embryonic development, morphogenesis, tissue repair, and regeneration. In addition to ability to inhibit metalloproteinases, TIMPs are involved in multiple biological functions such as cell proliferation, apoptosis, and synaptic plasticity, many of which are independent of MMP inhibitory activity [119]. Notably in this regard, TIMP2 expression in plasma and hippocampus decreases with age in mice [120]. Interestingly, Castellano et al. reported that human umbilical cord plasma enriched with TIMP2 improved hippocampal function, and systemic supplementation with TIMP2 increase synaptic plasticity and cognition in aged mice. On the basis of these findings, they suggested that plasma TIMP2 reverses age-related neuronal dysfunction. However, little is known regarding the molecular mechanism underlying this effect.

### SPARCL1 and THBS4

Ever since parabiosis experiments revealed that blood from young animals could rejuvenate neural function in old mice, many scientists used a wide range of approaches to identify rejuvenating factors in the blood. In a mass spectrometry study of plasma from young and old mice, Gan et al. found a series of proteins enriched in young or old blood [121]. Among the proteins they identified, SPARC-like protein 1 (SPARCL1) and thrombospondin-4 (THBS4) are abundant in young serum. Both proteins boost synaptic responses, synapse density, and dendritic branching in neurons transdifferentiated from human embryonic stem cells. Remarkably, these proteins are extracellular matrix-associated proteins and synaptogenic factors secreted from astrocytes. Astrocytes are essential for the function of the nervous system, as they regulate neurons by providing metabolic and trophic support [122]. THBS1 and 2 are astrocyte-secreted proteins that promotes synaptogenesis, whereas THBS4 enhance neurite adhesion and outgrowth [123]. SPARCL1 regulates CNS synaptogenesis [124]. Although THBS4 and SPARCL1, which are enriched in young mouse blood, increase synapse formation *in vitro*, their effects on brain rejuvenation in animal models remain unclear.

## CONCLUSIONS

Multiple studies have reported that young blood can reverse aspects of aging in various organs. Although young blood transfusion is effective for rejuvenation in aged mice, it remains unclear whether young blood transfusion into older people has clinical benefits. Young blood may contain multiple factors that contribute to the aging process, and studies using diverse approaches have identified several candidate anti-aging and pro-aging factors. The use of recombinant proteins as rejuvenating factors may enable the revitalization of aged organs in clinical application. Conboy’s team demonstrated that young blood by itself does not have positive effects on rejuvenating old tissues. [125]. Furthermore, the team found that replacing half of the blood plasma of old mice with a mixture of saline and albumin. is sufficient to rejuvenate the brain, liver, and muscle [126]. The authors suggested that young blood or rejuvenating factors are not required for rejuvenating effect, and removing pro-aging factors in old blood is effective way to rejuvenate old tissues. Thus, blockade of specific pro-aging factors or their receptors could contribute to treating age-related diseases. Although the mechanism by which these proteins act is far from being fully understood, multiple studies have demonstrated that these factors play important roles in age-related diseases, and may therefore have clinical applications in the future.
